# Effect of nintedanib in patients with progressive pulmonary fibrosis associated with rheumatoid arthritis: data from the INBUILD trial

**DOI:** 10.1007/s10067-023-06623-7

**Published:** 2023-05-20

**Authors:** Eric L. Matteson, Martin Aringer, Gerd R. Burmester, Heiko Mueller, Lizette Moros, Martin Kolb

**Affiliations:** 1 Division of Rheumatology, Mayo Clinic College of Medicine and Science, Rochester, MN USA; 2 Rheumatology, Medicine III, University Medical Center & Faculty of Medicine, TU Dresden, Dresden, Germany; 3 Department of Rheumatology and Clinical Immunology, Charité – Universitätsmedizin Berlin, Berlin, Germany; 4 Boehringer Ingelheim Pharma GmbH & Co. KG, Ingelheim am Rhein, Germany; 5 Boehringer Ingelheim International GmbH, Ingelheim am Rhein, Germany; 6 Department of Medicine, McMaster University and St. Joseph’s Healthcare, Hamilton, Ontario Canada

**Keywords:** Adverse drug event, Arthritis rheumatoid, Clinical trial, Interstitial lung disease, Pulmonary fibrosis

## Abstract

**Objectives:**

Some patients with rheumatoid arthritis develop interstitial lung disease (RA-ILD) that develops into progressive pulmonary fibrosis. We assessed the efficacy and safety of nintedanib versus placebo in patients with progressive RA-ILD in the INBUILD trial.

**Methods:**

The INBUILD trial enrolled patients with fibrosing ILD (reticular abnormality with traction bronchiectasis, with or without honeycombing) on high-resolution computed tomography of >10% extent. Patients had shown progression of pulmonary fibrosis within the prior 24 months, despite management in clinical practice. Subjects were randomised to receive nintedanib or placebo.

**Results:**

In the subgroup of 89 patients with RA-ILD, the rate of decline in FVC over 52 weeks was −82.6 mL/year in the nintedanib group versus −199.3 mL/year in the placebo group (difference 116.7 mL/year [95% CI 7.4, 226.1]; nominal *p* = 0.037). The most frequent adverse event was diarrhoea, which was reported in 61.9% and 27.7% of patients in the nintedanib and placebo groups, respectively, over the whole trial (median exposure: 17.4 months). Adverse events led to permanent discontinuation of trial drug in 23.8% and 17.0% of subjects in the nintedanib and placebo groups, respectively.

**Conclusions:**

In the INBUILD trial, nintedanib slowed the decline in FVC in patients with progressive fibrosing RA-ILD, with adverse events that were largely manageable. The efficacy and safety of nintedanib in these patients were consistent with the overall trial population. A graphical abstract is available at: https://www.globalmedcomms.com/respiratory/INBUILD_RA-ILD.

**Table:** 

**Key Points** • *In patients with rheumatoid arthritis and progressive pulmonary fibrosis, nintedanib reduced the rate of decline in forced vital capacity (mL/year) over 52 weeks by 59% compared with placebo.* • *The adverse event profile of nintedanib was consistent with that previously observed in patients with pulmonary fibrosis, characterised mainly by diarrhoea.* • *The effect of nintedanib on slowing decline in forced vital capacity, and its safety profile, appeared to be consistent between patients who were taking DMARDs and/or glucocorticoids at baseline and the overall population of patients with rheumatoid arthritis and progressive pulmonary fibrosis.*

**Supplementary Information:**

The online version contains supplementary material available at 10.1007/s10067-023-06623-7.

## Introduction

Interstitial lung disease (ILD) may occur as a manifestation of rheumatoid arthritis (RA) [1, 2]. High-resolution computed tomography (HRCT) scans from patients with RA-ILD commonly show fibrotic as well as inflammatory features [3]. The clinical course of RA-ILD is variable and difficult to predict [4, 5]. Some patients with RA-ILD develop progressive pulmonary fibrosis characterised by increasing radiological fibrosis, decline in lung function, worsening symptoms, and early mortality [4–8]. It has been estimated that ILD is a contributor to mortality in up to 6.8% of women and 9.8% of men with RA [2]. As in patients with other ILDs, loss of forced vital capacity (FVC) is a predictor of mortality in patients with RA-ILD [9–11].

Disease-modifying anti-rheumatic drugs (DMARDs) and glucocorticoids are the standard of care for RA [12], but there is no evidence from randomised controlled trials that they slow the progression of pulmonary fibrosis. Nintedanib is a tyrosine kinase inhibitor licensed for the treatment of idiopathic pulmonary fibrosis (IPF), fibrosing ILD associated with systemic sclerosis (SSc), and progressive fibrosing ILDs of any aetiology. Pre-clinical studies have shown that nintedanib has anti-fibrotic and anti-inflammatory effects that slow the progression of pulmonary fibrosis, including inhibiting the release of pro-fibrotic and pro-inflammatory mediators, the migration and differentiation of fibroblasts, and the accumulation of extracellular matrix [13]. Nintedanib was given a conditional recommendation for use in patients with progressive pulmonary fibrosis who have failed “standard management” for ILD in international management guidelines [14]. In the randomised placebo-controlled INBUILD trial in patients with progressive fibrosing ILDs other than IPF, nintedanib slowed the progression of pulmonary fibrosis, with an adverse event profile characterised mainly by gastrointestinal events [15–17]. No heterogeneity was detected in the effect of nintedanib on reducing the rate of decline in FVC across diagnostic subgroups [15, 18, 19]. Here, we assessed the efficacy and safety of nintedanib in patients with progressive RA-ILD in the INBUILD trial.

## Methods

### Trial design

The INBUILD trial (NCT02999178) was a randomised, double-blind, placebo-controlled trial conducted in 15 countries [15, 16]. The trial was conducted in accordance with the protocol, the principles of the Declaration of Helsinki, and the Harmonised Tripartite Guideline for Good Clinical Practice from the International Conference on Harmonisation and was approved by local authorities. Written informed consent was obtained from all patients before study entry. The trial design has been described and the protocol is publicly available [15]. Briefly, patients had an ILD other than IPF with reticular abnormality with traction bronchiectasis (with or without honeycombing) of >10% extent on HRCT, FVC ≥45% predicted and diffusing capacity of the lungs for carbon monoxide (DLco) ≥30–<80% predicted. Patients met ≥1 of the following criteria for progression of pulmonary fibrosis within the prior 24 months, despite management deemed appropriate in clinical practice: relative decline in FVC ≥10% predicted; relative decline in FVC ≥5–<10% predicted and worsened respiratory symptoms; relative decline in FVC ≥5–<10% predicted and increased extent of fibrosis on HRCT; worsened respiratory symptoms and increased extent of fibrosis on HRCT. Azathioprine, cyclosporine, mycophenolate mofetil, tacrolimus, oral glucocorticoids >20 mg/day, or the combination of oral glucocorticoids, azathioprine, and N-acetylcysteine was not permitted ≤4 weeks prior to randomisation; cyclophosphamide was not permitted ≤8 weeks prior to randomisation; rituximab was not permitted ≤6 months prior to randomisation; there was no limit on the use of stable doses of other biologic or non-biologic DMARDs. Use of glucocorticoids at a dose of ≤20 mg/day prednisone or equivalent was permitted. The immunomodulatory therapies that were not permitted at randomisation could be initiated after 6 months in patients with deterioration of ILD or connective tissue disease, but use of nintedanib (other than as trial drug) and pirfenidone was prohibited.

Patients were randomised to receive nintedanib 150 mg twice daily (bid) or placebo, stratified by fibrotic pattern on HRCT (usual interstitial pneumonia [UIP]-like fibrotic pattern or other fibrotic patterns [described in 15]). Treatment interruptions (for ≤4 weeks for adverse events considered related to trial medication or ≤8 weeks for other adverse events) and dose reductions to 100 mg bid were used to manage adverse events. After resolution of the adverse event, nintedanib could be reintroduced and/or the dose increased back to 150 mg bid. The trial consisted of two parts. Part A comprised 52 weeks of treatment. Part B was a variable period beyond week 52 during which patients continued to receive blinded treatment until all patients had completed the trial. Patients who discontinued treatment were asked to attend all visits as planned, including an end-of-treatment visit and a follow-up visit 4 weeks later. The final database lock took place after all patients had completed the follow-up visit or had entered the open-label extension study, INBUILD-ON (NCT03820726). The data available at final database lock comprised the data from the whole trial.

### Outcomes

We assessed the rate of decline in FVC (mL/year) over 52 weeks in all patients with RA-ILD, in subgroups of patients with RA-ILD based on high sensitivity C-reactive protein (hs-CRP) at baseline (<1 vs ≥1 mg/L; <3 vs ≥3 mg/L) and in patients with RA-ILD taking DMARDs and/or glucocorticoids at baseline. DMARDs were identified based on the WHO Drug Dictionary (version 19.MAR) standardised drug grouping with the addition of baricitinib and the exclusion of denosumab. Glucocorticoids were identified based on the WHO Drug Dictionary standardised drug grouping.

We report the proportions of patients with RA-ILD with absolute and relative declines from baseline in FVC % predicted >10% at week 52. We also analysed the times to first acute exacerbation (defined in [15]) or death, first non-elective hospitalisation or death, first non-elective respiratory hospitalisation or death, progression of ILD (defined as an absolute decline from baseline in FVC % predicted ≥ 10%) or death, and death using data from the whole trial. Adverse events reported over the whole trial, irrespective of causality, were coded using preferred terms in the Medical Dictionary for Regulatory Activities (MedDRA) version 22.0 and are presented descriptively.

### Analyses

Analyses were based on data from patients who received ≥1 dose of trial drug. The rate of decline in FVC (mL/year) over 52 weeks was analysed in patients with RA-ILD using a random coefficient regression model (with random slopes and intercepts) including baseline FVC (mL), HRCT pattern (UIP-like fibrotic pattern or other fibrotic patterns) and treatment, and treatment-by-time and baseline-by-time interactions. Subgroup analyses used the same model but with interaction terms for baseline-by-time, treatment-by-subgroup, and treatment-by-subgroup-by-time interaction. In subgroups by CRP at baseline, the interaction *p*-value (based on an *F*-test) was an indicator of potential heterogeneity of the effect of nintedanib versus placebo between the subgroups. The proportions of patients with absolute and relative declines in FVC >10% predicted at week 52 were analysed using logistic regression. Missing values at week 52 were imputed using multiple imputation. Cox proportional hazards models (stratified by HRCT pattern) with terms for treatment were used to derive hazard ratios (HRs) and 95% confidence intervals (CIs). *P*-values were calculated based on a log-rank test stratified by HRCT pattern. Analyses were not adjusted for multiplicity.

## Results

### Patients

Of 663 patients in the INBUILD trial, 89 (13.4%) had RA-ILD. The RA diagnosis was confirmed by a rheumatologist in 83 of the 84 patients for whom these data were available. The baseline characteristics of the patients with RA-ILD have been described [19]. In summary, mean (SD) age was 66.9 (9.6) years, 60.7% were male, and 86.5% had a UIP-like fibrotic pattern on HRCT. Mean (SD) FVC was 71.5 (16.2) % predicted and mean (SD) DLco was 47.7 (15.6) % predicted. Mean (SD) times since diagnosis of RA and RA-ILD were 9.9 (9.4) years and 3.6 (3.2) years. Among patients with available CRP measurements, mean (SD) hs-CRP was 13.7 (22.5) mg/L; 64.7% of patients had hs-CRP ≥3.0 mg/L. A total of 21.3% were taking biologic DMARDs, 53.9% non-biologic DMARDs, and 73.0% glucocorticoids (Online Resource 1). The most common comorbidities were hypertension and gastro-oesophageal reflux disease (Online Resource 2).

### Exposure to medications

Median exposure to trial drug over the whole trial was 17.4 months in both groups. Based on medications taken at baseline, during treatment with trial drug, or following discontinuation of trial drug, the immunomodulatory therapies that were restricted at baseline were taken by higher proportions of patients in the placebo group than in the nintedanib group (Online Resource 3).

### Decline in FVC

**Fig. 1:**
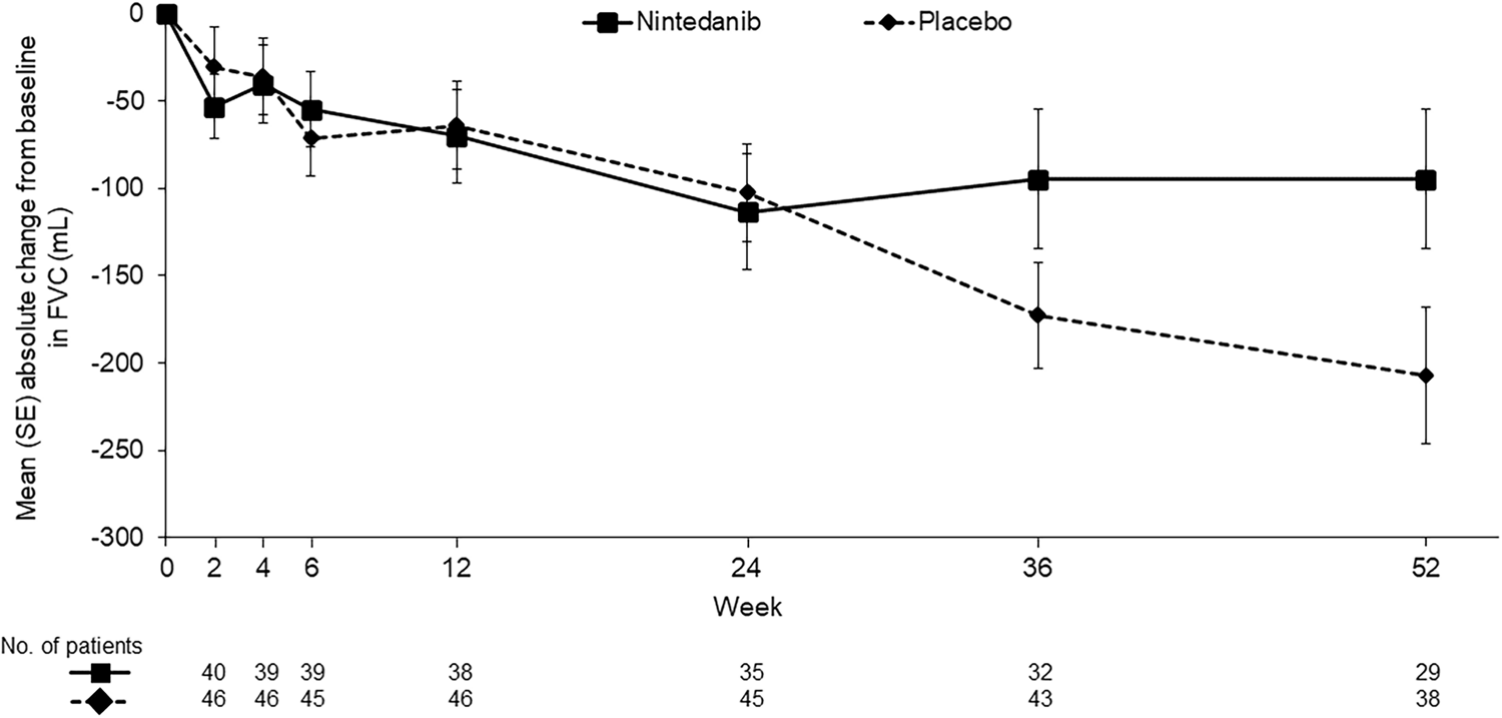
Change in forced vital capacity (FVC) (mL) over 52 weeks in patients with RA-ILD in the INBUILD trial. *RA-ILD* rheumatoid arthritis-associated interstitial lung disease

**Fig. 2:**
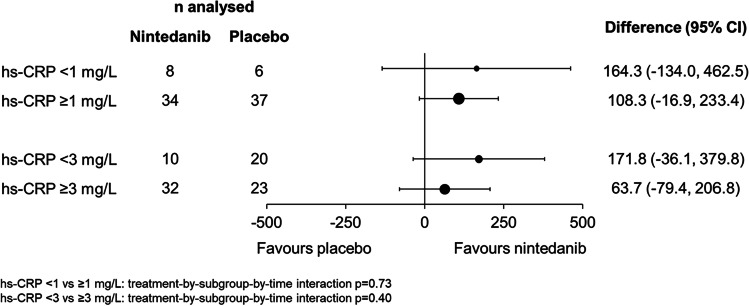
Rate of decline in FVC (mL/year) over 52 weeks in subgroups of patients with RA-ILD in the INBUILD trial by high sensitivity C-reactive protein (hs-CRP) at baseline. *RA-ILD* rheumatoid arthritis-associated interstitial lung disease

In this evaluation of patients with RA-ILD, the adjusted annual rate of decline in FVC over 52 weeks was −82.6 mL/year in the nintedanib group versus −199.3 mL/year in the placebo group (difference 116.7 mL/year [95% CI 7.4, 226.1]; nominal *p* = 0.037) (Table 1). Observed changes from baseline in FVC (mL) showed separation between treatment groups from week 24 (Fig. 1). Among these patients with RA-ILD, no heterogeneity was detected in the effect of nintedanib versus placebo on the rate of decline in FVC (mL/year) across subgroups by CRP at baseline (Fig. 2). Among patients with RA-ILD taking DMARDs and/or glucocorticoids at baseline, the adjusted annual rate of decline in FVC over 52 weeks was −90.4 mL/year in the nintedanib group (*n* = 39) versus −228.3 mL/year in the placebo group (*n* = 40) (difference 137.9 mL/year [95% CI 23.4, 252.5]). Smaller proportions of patients in the nintedanib group than in the placebo group had absolute and relative declines in FVC % predicted >10% at week 52 (Table 1).

**Table 1:** Efficacy endpoints in patients with RA-ILD in the INBUILD trial

	Nintedanib (*n* = 42)	Placebo (*n* = 47)
Rate of decline in FVC (mL/year) over 52 weeks		
Rate of decline in FVC (mL/year) over 52 weeks, adjusted mean (SE)	−82.6 (41.3)	−199.3 (36.2)
Difference (95% CI)	116.7 (7.4, 226.1)	
Nominal *p*-value	0.037	
Absolute and relative declines in FVC at week 52		
Relative decline in FVC >10% predicted at week 52, *n* (%)	12 (28.6)	20 (42.6)
Odds ratio (95% CI)	0.48 (0.19 1.25)	
Absolute decline in FVC >10% predicted at week 52, *n* (%)	5 (11.9)	15 (31.9)
Odds ratio (95% CI)	0.31 (0.10, 1.02)	
Time to event endpoints over the whole trial		
Acute exacerbation of ILD or death, *n* (%)	8 (19.0)	15 (31.9)
Hazard ratio (95% CI)	0.54 (0.23, 1.28)	
Nominal *p*-value	0.16	
Hospitalisation or death, *n* (%)	27 (64.3)	26 (55.3)
Hazard ratio (95% CI)	1.36 (0.79, 2.34)	
Nominal *p*-value	0.26	
Respiratory hospitalisation or death, *n* (%)	18 (42.9)	22 (46.8)
Hazard ratio (95% CI)	0.87 (0.46, 1.62)	
Nominal *p*-value	0.65	
Progression of ILD^a^ or death, *n* (%)	19 (45.2)	29 (61.7)
Hazard ratio (95% CI)	0.63 (0.35, 1.13)	
Nominal *p*-value	0.12	
Death, *n* (%)	7 (16.7)	9 (19.1)
Hazard ratio (95% CI)	0.86 (0.32, 2.31)	
Nominal *p*-value	0.76	

^a^Defined as absolute decline from baseline in FVC ≥ 10% predicted. *FVC* forced vital capacity, *ILD* interstitial lung disease, *RA-ILD* rheumatoid arthritis-associated interstitial lung disease

### Acute exacerbations, hospitalisations, progression of ILD, and death

Time to event endpoints over the whole trial are shown in Table 1 and Online Resource 4. Acute exacerbation of ILD or death occurred in 8 patients (19.0%) in the nintedanib group and 15 (31.9%) in the placebo group (HR 0.54 [95% CI 0.3, 1.28]; nominal *p* = 0.16). Hospitalisation or death occurred in 27 patients (64.3%) in the nintedanib group and 26 patients (55.3%) in the placebo group (HR 1.36 [95% CI 0.79, 2.34]; nominal *p* = 0.26), and respiratory hospitalisation or death in 18 patients (42.9%) in the nintedanib group and 22 patients (46.8%) in the placebo group (HR 0.87 [95% CI 0.46, 1.62]; nominal *p* = 0.65). Progression of ILD or death occurred in 19 patients (45.2%) in the nintedanib group and 29 patients (61.7%) in the placebo group (HR 0.63 [95% CI 0.35, 1.13]; nominal *p* = 0.12). Deaths occurred in 7 patients (16.7%) in the nintedanib group and 9 patients (19.2%) in the placebo group (HR 0.86 [95% CI 0.32, 2.31]; nominal *p* = 0.76).

### Safety and tolerability

The most common adverse event reported in these patients with RA-ILD was diarrhoea, which was reported in 61.9% of patients in the nintedanib group and 27.7% of patients in the placebo group (Table 2). Diarrhoea occurred at a rate of 118 and 26 events per 100 patient-years in the nintedanib and placebo groups, respectively. Nausea was reported in 21.4% of patients in the nintedanib group and 12.8% of patients in the placebo group. Serious adverse events were reported in similar proportions of patients in the nintedanib and placebo groups (61.9% and 61.7%, respectively). Adverse events led to permanent dose reduction in 21.4% of patients in the nintedanib group and none in the placebo group. Adverse events led to treatment discontinuation in 23.8% of patients in the nintedanib group and 17.0% of patients in the placebo group. The most frequent adverse event leading to discontinuation of nintedanib was an increase in alanine aminotransferase, which led to discontinuation in 7.1% of patients. Gastrointestinal adverse events led to discontinuation of nintedanib in two (4.8%) patients.

**Table 2:** Adverse events in patients with RA-ILD in the INBUILD trial

	Nintedanib (*n* = 42)		Placebo (*n* = 47)	
	*n* (%)	Rate per 100 patient-years	*n* (%)	Rate per 100 patient-years
Any adverse event	42 (100.0)	1124.7	45 (95.7)	425.4
Most frequent adverse events^a^				
Diarrhoea	26 (61.9)	118.0	13 (27.7)	26.0
Bronchitis	9 (21.4)	21.3	14 (29.8)	24.9
Pneumonia	10 (23.8)	19.9	6 (12.8)	9.4
Nausea	9 (21.4)	21.0	6 (12.8)	10.3
Dyspnoea	8 (19.0)	18.1	6 (12.8)	9.6
Nasopharyngitis	4 (9.5)	7.9	7 (14.9)	12.3
Constipation	6 (14.3)	12.8	4 (8.5)	6.4
Arthralgia	5 (11.9)	10.5	5 (10.6)	8.2
Vomiting	5 (11.9)	10.9	4 (8.5)	6.6
Progression of ILD^b^	2 (4.8)	3.8	7 (14.9)	11.3
Abdominal pain	5 (11.9)	11.0	3 (6.4)	4.6
Alanine aminotransferase increased	6 (14.3)	12.5	1 (2.1)	1.5
Back pain	5 (11.9)	10.0	2 (4.3)	3.1
Decreased appetite	5 (11.9)	10.7	1 (2.1)	1.5
Urinary tract infection	5 (11.9)	10.6	1 (2.1)	1.5
Abdominal pain upper	5 (11.9)	10.6	1 (2.1)	1.6
Serious adverse event^c^	26 (61.9)	81.7	29 (61.7)	60.2
Adverse event leading to permanent dose reduction	9 (21.4)	21.5	0	0
Adverse event leading to treatment discontinuation	10 (23.8)	19.2	8 (17.0)	12.2
Most frequent adverse events leading to treatment discontinuation^d^				
Alanine aminotransferase increased	3 (7.1)	5.7	0	0
Diarrhoea	2 (4.8)	3.8	0	0
Aspartate aminotransferase increased	2 (4.8)	3.8	0	0

Based on adverse events reported between first trial drug intake and 28 days after last trial drug intake. Median exposure to trial drug was 17.4 months in both groups. Adverse events were coded based on preferred terms in MedDRA version 22.0. ^a^Adverse events with a rate >10 per 100 patient-years in either treatment group are shown. ^b^Based on MedDRA preferred term “interstitial lung disease”. ^c^Adverse event that resulted in death, was life-threatening, resulted in hospitalisation or prolongation of hospitalisation, resulted in persistent or clinically significant disability or incapacity, was a congenital anomaly or birth defect, or was deemed to be serious for any other reason. ^d^Adverse events that led to treatment discontinuation with a rate >2 per 100 patient-years in either treatment group are shown. *ILD*, interstitial lung disease; *RA-ILD*, rheumatoid arthritis-associated interstitial lung disease; *MedDRA*, Medical Dictionary for Regulatory Activities

## Discussion

These data from the INBUILD trial show that nintedanib reduced the rate of decline in FVC over 52 weeks in patients with progressive fibrosing RA-ILD by 59% compared with placebo, similar to the relative treatment effect observed in the overall trial population [15], in patients with SSc-ILD [20] and in patients with IPF [21]. A recent meta-analysis of the effect of nintedanib on the rate of FVC decline across ILDs confirmed that there was no evidence of heterogeneity [22]. These findings provide further support for the hypothesis that the progressive pulmonary fibrosis that develops in a proportion of patients with various ILDs develops via common pathological pathways and that nintedanib inhibits pathways fundamental to the progression of pulmonary fibrosis [13, 23]. The later separation of the curves of change in FVC in the RA-ILD subgroup of the INBUILD trial than in the overall trial population may be a reflection of the relatively small size of this subgroup.

Decline in FVC in patients with ILDs is reflective of disease progression and is associated with mortality [9–11, 24, 25]. The patients with RA-ILD who participated in the INBUILD trial showed significant loss of lung function over 52 weeks, with a decline of over 200 mL of FVC observed in the placebo group. This highlights the importance of prompt identification and treatment of patients with RA who have progressive pulmonary fibrosis. Although some predictors of progressive RA-ILD have been identified [3, 6, 9, 10, 26, 27], the course of RA-ILD in an individual patient remains largely unpredictable, making regular monitoring of lung function an important part of the care of these patients. In clinical practice, progression of fibrosing ILD is usually assessed based on lung function tests performed every 3 to 6 months [28, 29].

CRP is a marker of inflammation [30]. In a prospective study of patients with SSc-ILD, elevated CRP was associated with greater decline in FVC and shorter survival [31], but it is unclear whether elevated CRP is associated with progression of RA-ILD. We found no heterogeneity in the effect of nintedanib between patients with differing CRP levels at baseline, consistent with findings in patients with SSc-ILD [32].

Acute exacerbations associated with high morbidity and mortality are a feature of the natural history of IPF [33] and have also been reported in patients with RA-ILD [8, 9, 34–36] and other autoimmune disease-related ILDs [37]. Over the whole INBUILD trial, 32% of the patients with progressive fibrosing RA-ILD who received placebo had an acute exacerbation of ILD or died. Treatment with nintedanib was associated with a numerically reduced risk of acute exacerbation of ILD or death (HR 0.54). This trend may be of clinical relevance given the lack of effective treatments for acute exacerbations of ILD.

The adverse event profile of nintedanib in patients with RA-ILD in the INBUILD trial was consistent with observations in the overall trial population [15–17] and in the subgroup of patients with autoimmune disease-related ILDs [19] as well as in patients with SSc-ILD [20, 38] and IPF [21, 39]. Diarrhoea was the most common adverse event, but fewer than 5% of the patients with RA-ILD discontinued nintedanib due to diarrhoea. In clinical practice, management of adverse events using symptomatic therapies and dose adjustment is important to minimise their impact and help patients remain on therapy.

DMARDs are an important part of the management of patients with RA, according to international management guidelines [12, 40] and most patients with RA are taking DMARDs and/or glucocorticoids [40]. There is little evidence to suggest that the use of DMARDs is associated with worsening of RA-ILD, although tumour necrosis factor (TNF)-alpha inhibitors may be associated with higher mortality than other DMARDs [41, 42]. Among patients with RA-ILD, the effect of nintedanib on reducing decline in FVC in patients who were taking DMARDs and/or glucocorticoids at baseline was similar to that observed in all patients with RA-ILD. Previous analyses of data from the overall INBUILD trial population supported this observation and suggested that the introduction of restricted immunomodulatory therapies during the trial had no impact on the effect of nintedanib [43]. However, it should be borne in mind that patients were not randomised by use of therapies other than nintedanib. Further research is needed into the effects of therapy with nintedanib and immunomodulators in patients with autoimmune disease-related ILDs.

Strengths of our analyses include the randomised placebo-controlled trial design, with standardised reporting of outcomes and adverse events. Limitations include that the INBUILD trial was not designed or powered to study individual ILDs and the number of patients with RA-ILD was small, limiting the potential for subgroup analyses including those based on concomitant medication use. There are no adequately validated patient-reported outcomes for assessing changes in symptoms or quality of life due to worsening of RA-ILD. Data on RA disease activity were not collected.

## Conclusions

In the INBUILD trial, nintedanib slowed the rate of decline in FVC in patients with progressive fibrosing RA-ILD, with adverse events that were largely manageable. The efficacy and safety of nintedanib in these patients were consistent with the overall trial population.

## Supplementary information

ESM 1. (DOCX 245 kb)

ESM 2. (MP4 20280 kb)

## Data Availability

To ensure independent interpretation of clinical study results and enable authors to fulfill their role and obligations under the ICMJE criteria, Boehringer Ingelheim grants all external authors access to relevant clinical study data. In adherence with the Boehringer Ingelheim Policy on Transparency and Publication of Clinical Study Data, scientific and medical researchers can request access to clinical study data after publication of the primary manuscript in a peer-reviewed journal, regulatory activities are complete and other criteria are met. Researchers should use https://vivli.org/ to request access to study data and visit https://www.mystudywindow.com/msw/datasharing for further information.
