# Supplementary material for: Effect of nintedanib in patients with progressive pulmonary fibrosis associated with rheumatoid arthritis: data from the INBUILD trial
Source: Clin Rheumatol. 2023 May 20;42(9):2311–9. doi: 10.1007/s10067-023-06623-7 (PMC10412475; doi:10.1007/s10067-023-06623-7)

**Effect of nintedanib in patients with progressive pulmonary fibrosis associated with rheumatoid arthritis: data from the INBUILD trial (Matteson EL et al)**

**SUPPLEMENTARY INFORMATION**

**Online Resource 1** Immunomodulatory medications taken at baseline in patients with RA-ILD in the INBUILD trial.

| **≥1 immunomodulatory medication** | **79 (88.8)** |
| --- | --- |
| **Biologic DMARDs** | **19 (21.3)** |
| Abatacept | 6 (6.7) |
| Etanercept | 4 (4.5) |
| Adalimumab | 3 (3.4) |
| Tocilizumab | 3 (3.4) |
| Infliximab | 2 (2.2) |
| Rituximab | 1 (1.1) |
| **Non-biologic DMARDs** | **48 (53.9)** |
| Hydroxychloroquine/hydroxychloroquine sulphate | 18 (20.2) |
| Leflunomide | 15 (16.9) |
| Methotrexate/methotrexate sodium | 13 (14.6) |
| Sulfasalazine | 10 (11.2) |
| Bucillamine | 1 (1.1) |
| Chloroquine phosphate | 1 (1.1) |
| Iguratimod | 1 (1.1) |
| **Glucocorticoids^a^** | **65 (73.0)** |
| Prednisone | 33 (37.1) |
| Prednisolone | 23 (25.8) |
| Methylprednisolone | 4 (4.5) |
| Meprednisone | 2 (2.2) |
| Cortisone acetate | 1 (1.1) |
| Deflazacort | 1 (1.1) |
| Triamcinolone acetonide | 1 (1.1) |

Data are n (%) of patients with RA-ILD (n=89) based on customised drug groupings (bold) and preferred names (non-bold). ^a^Based on customised drug grouping “corticosteroids”; restricted to oral, intravenous, intravenous bolus, intravenous drip, or intramuscular administration. *DMARDs* disease-modifying anti-rheumatic drugs, *RA-ILD* rheumatoid arthritis-associated interstitial lung disease.

**Online Resource 2** Comorbidities at baseline in patients with RA-ILD in the INBUILD trial.

| Hypertension | 48 (53.9) |
| --- | --- |
| Gastro-oesophageal reflux disease | 22 (24.7) |
| Menopause | 18 (20.2) |
| Hyperlipidaemia | 16 (18.0) |
| Diabetes mellitus | 12 (13.5) |
| Dyslipidaemia | 11 (12.4) |
| Benign prostatic hyperplasia | 10 (11.2) |
| Osteoporosis | 10 (11.2) |
| Sleep apnoea syndrome | 10 (11.2) |
| Post-menopause | 9 (10.1) |
| Asthma | 9 (10.1) |
| Cataract | 9 (10.1) |
| Rhinitis allergic | 9 (10.1) |

Data are n (%) of patients with RA-ILD (n=89) based on preferred terms in MedDRA version 22.0. Comorbidities reported in ≥10% of patients are shown. *MedDRA*, Medical Dictionary for Regulatory Activities*, RA-ILD,* rheumatoid arthritis-associated interstitial lung disease.

**Online Resource 3** Restricted or prohibited immunomodulatory or antifibrotic therapies taken at baseline, during treatment with trial drug, or following discontinuation of trial drug in patients with RA-ILD in the INBUILD trial.

|  | **Nintedanib (n=42)** | **Placebo**  **(n=47)** |
| --- | --- | --- |
| ≥1 restricted or prohibited therapy | 14 (33.3) | 21 (44.7) |
| Glucocorticoids | 12 (28.6) | 20 (42.6) |
| Mycophenolate mofetil | 0 | 3 (6.4) |
| Rituximab | 2 (4.8) | 1 (2.1) |
| Cyclophosphamide | 0 | 2 (4.3) |
| Tacrolimus | 0 | 1 (2.1) |
| Nintedanib | 0 | 1 (2.1) |
| Azathioprine | 1 (2.4) | 0 |
| Pirfenidone | 1 (2.4) | 0 |

Data are n (%) of patients who took ≥1 such therapy at baseline, during treatment with trial drug, and/or following discontinuation of trial drug for any duration. Glucocorticoids were based on the customised drug grouping “corticosteroids”; for other therapies, preferred names are shown. Glucocorticoids were counted as restricted therapies if used at dose of >20 mg/day prednisone or equivalent via oral, intravenous, intravenous bolus, intravenous drip, or intramuscular administration. *RA-ILD* rheumatoid arthritis-associated interstitial lung disease.

**Online Resource 4** Kaplan-Meier estimates of time to (**A**) acute exacerbation of ILD or death, (**B**) hospitalisation or death, (**C**) respiratory hospitalisation or death, (**D**) progression of ILD (absolute decline in FVC % predicted ≥10%) or death and (**E**) death in patients with RA-ILD in the INBUILD trial. *FVC* forced vital capacity, *ILD*, interstitial lung disease, *RA-ILD* rheumatoid arthritis-associated interstitial lung disease

**A**


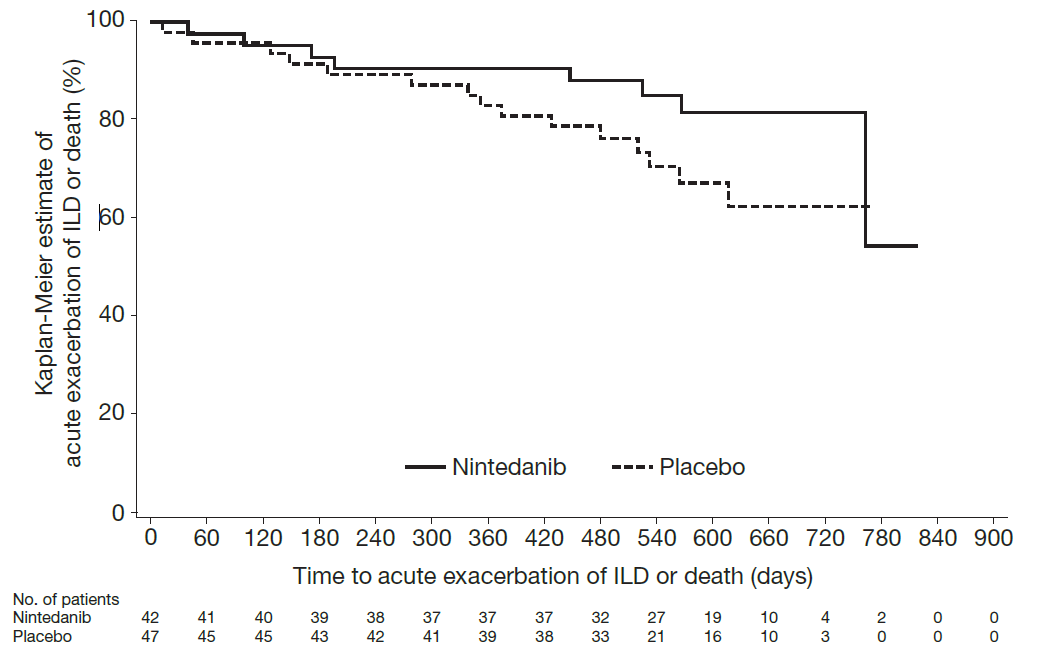


**B**


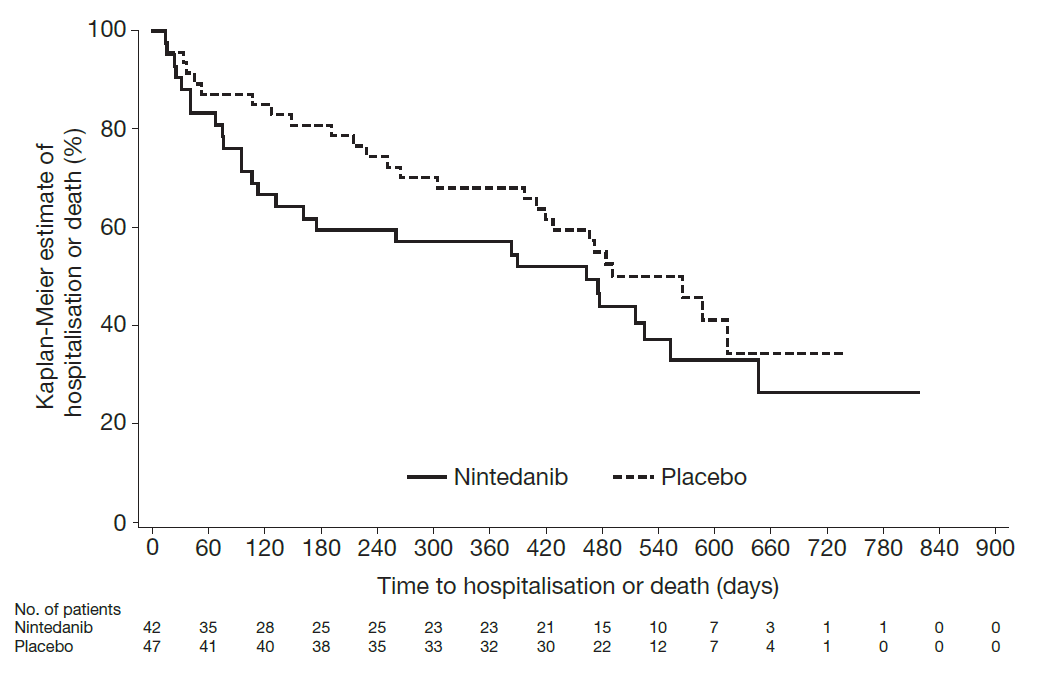


**C**


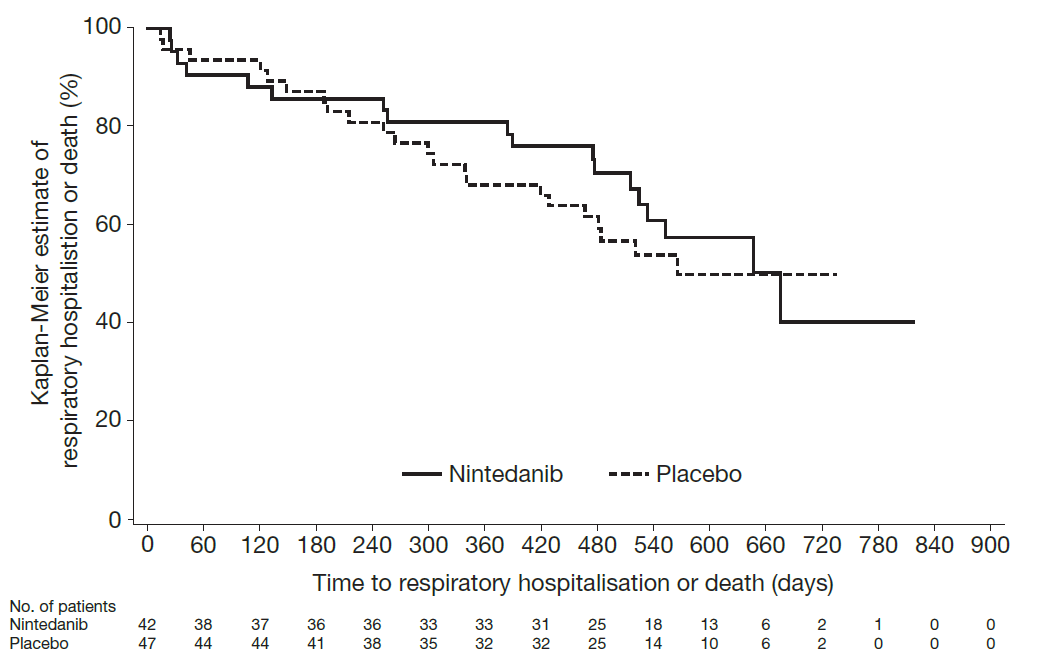


**D**


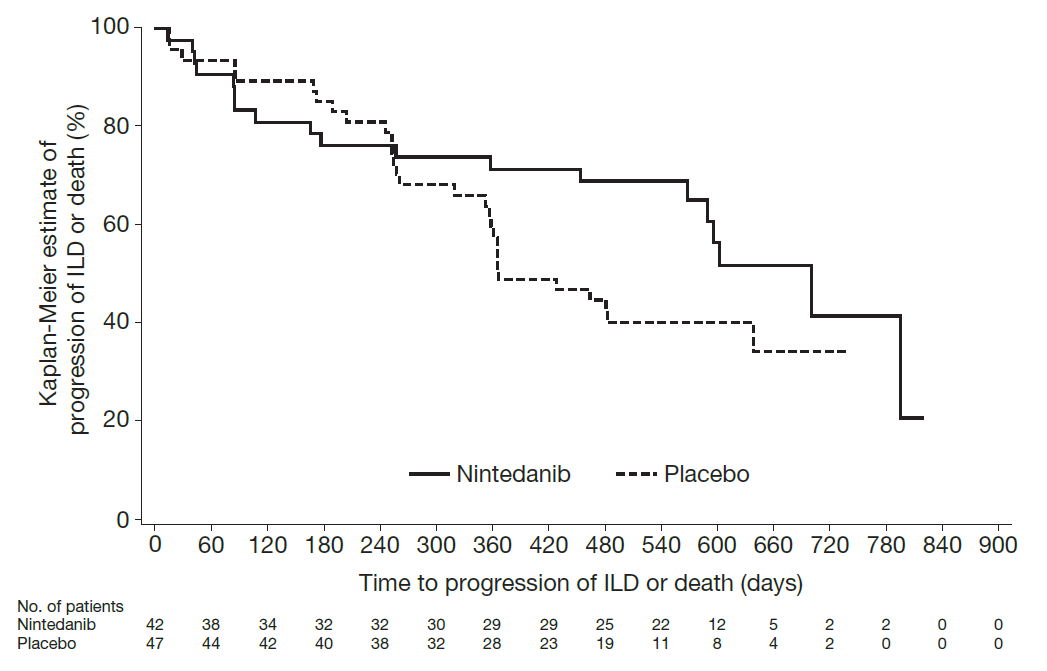


**E**


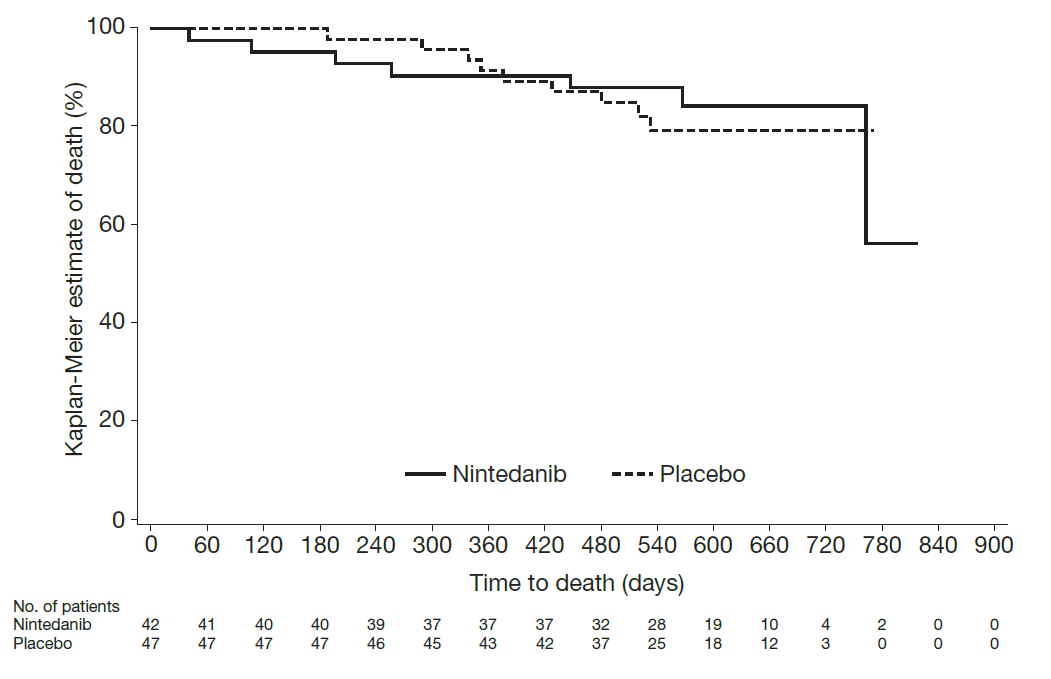

Supplement: Supplementary file 1 — (DOCX 245 kb) [file 10067_2023_6623_MOESM1_ESM.docx]
